# The Ibr‐7 derivative of ibrutinib exhibits enhanced cytotoxicity against non‐small cell lung cancer cells via targeting of mTORC1/S6 signaling

**DOI:** 10.1002/1878-0261.12454

**Published:** 2019-02-22

**Authors:** Bo Zhang, Linling Wang, Qi Zhang, Youyou Yan, Hong Jiang, Runlei Hu, Xinglu Zhou, Xingguo Liu, Jianguo Feng, Nengming Lin

**Affiliations:** ^1^ Translational Medicine Research Center Affiliated Hangzhou First People's Hospital Zhejiang University School of Medicine Hangzhou China; ^2^ Affiliated Hangzhou First People's Hospital Zhejiang Chinese Medical University Hangzhou China; ^3^ Shaoxing Hospital of Traditional Chinese Medicine Shaoxing China; ^4^ Department of Thoracic Surgery Affiliated Hangzhou First People's Hospital Zhejiang University School of Medicine Hangzhou China; ^5^ Hangzhou Hertz Pharmaceutical Co. China; ^6^ Cancer Research Institute Zhejiang Cancer Hospital Hangzhou China

**Keywords:** ABT‐199, Ibr‐7, ibrutinib, mTORC1, S6, non‐small cell lung cancer

## Abstract

Ibrutinib is a small molecule drug that targets Bruton's tyrosine kinase in B‐cell malignancies and is highly efficient at killing mantle cell lymphoma and chronic lymphocytic leukemia. However, the anti‐cancer activity of ibrutinib against solid tumors, such as non‐small cell lung cancer (NSCLC), remains low. To improve the cytotoxicity of ibrutinib towards lung cancer, we synthesized a series of ibrutinib derivatives, of which Ibr‐7 exhibited superior anti‐cancer activity to ibrutinib, especially against epithelial growth factor receptor (EGFR) wild‐type NSCLC cell lines. Ibr‐7 was observed to dramatically suppress the mammalian target of Rapamycin complex 1 (mTORC1)/S6 signaling pathway, which is only slightly affected by ibrutinib, thus accounting for the superior anti‐cancer activity of Ibr‐7 towards NSCLC. Ibr‐7 was shown to overcome the elevation of Mcl‐1 caused by ABT‐199 mono‐treatment, and thus exhibited a significant synergistic effect when combined with ABT‐199. In conclusion, we used a molecular substitution method to generate a novel ibrutinib derivative, termed Ibr‐7, which exhibits enhanced anti‐cancer activity against NSCLC cells as compared with the parental compound.

**Abbreviations**
BCR: B cell receptor
BTK: Bruton's tyrosine kinase
CD‐DST: collagen gel droplet embedded 3D‐culture system
EGFR: epidermal growth factor receptor
FFPE: formalin‐fixed and paraffin‐embedded
LARP1: La‐related protein 1
mTOR: the mammalian target of rapamycin
NSCLC: non‐small cell lung cancer
PARP: Poly (ADP‐ribose) polymerase
SILAC: stable isotope labeling with amino acid
TKI: tyrosine kinase inhibitor
XIAP: X‐linked inhibitor of apoptosis protein

## 1 Introduction

Ibrutinib (PCI‐32765, IMBRUVICA®) is an orally available small molecule inhibitor that targets Bruton´s tyrosine kinase (BTK) to impair B cell receptor (BCR) signaling, thus stalling the development and maturation of B cells (Burger and Wiestner, 2018). Ibrutinib specifically binds to Cys481 in the ATP‐binding site, which is a conserved domain among other tyrosine kinases such as epidermal growth factor receptor (EGFR) or HER2 (Chen *et al*., 2016, 2018). It was initially found that ibrutinib exhibited potent antitumor effect against non‐small cell lung cancer (NSCLC) cells, but only those with EGFR‐mutant (L858R or 19Del) (Grabinski and Ewald, 2014; Gao *et al*., 2014; He *et al*., 2017). In screening various cancer cell lines, it was demonstrated that the growth inhibitory effect of ibrutinib against cancer cells was limited to blood cancer cells (Table S1). Due to the enormous differences of ibrutinib's antitumor efficacy between lymphoma and solid tumors, the undergoing clinical trials which apply ibrutinib as a single agent to treat solid tumors are facing an inevitable dilemma. Therefore, it is of great interest to improve the sensitivity of ibrutinib or its derivatives towards solid tumor cells.

In this study, we sought to exploit novel BTK inhibitors, aiming not only to enhance the antitumor potency to solid tumor cells but also to increase the kinase selectivity and reduce the risk of nonspecific covalent binding. To this end, we focused on replacement of the acrylamide and diphenyl ether using a bioisosterism strategy, affording compound Ibr‐7, which showed a 5‐ to 50‐fold increased cytotoxicity towards lung and pancreatic cancer cells. Moreover, the results of pharmacokinetics and hERG safety assay suggested that Ibr‐7 would be a more promising candidate for further studies (Table S3). To explore the underlying mechanisms of Ibr‐7 in lung cancer cells, SILAC assay was performed to obtain a general inhibitory spectrum on phosphorylated proteins. As distinct from ibrutinib, Ibr‐7 potently suppressed the phosphorylation of the mTORC1/S6 pathway. Taking advantage of this unique mechanism of action, Ibr‐7 could be applied to sensitize dramatically ABT‐199 via the synthetic inhibition of Mcl‐1 protein in NSCLC cells. In this study, Ibr‐7 exhibited its dual inhibitory activity towards EGFR and mTORC1/S6, and displaying enhanced cytotoxicity against NSCLC cells; these results will provide meaningful insights into the development of novel BTK inhibitors.

## 2 Materials and methods

### 2.1 Cell lines and reagents

The PC‐9, NCI‐H1975, A549 and NCI‐H460 cell lines were purchased from the Cell Bank of Type Culture Collection of the Chinese Academy of Sciences (Shanghai, China). All the cell lines were tested and authenticated utilizing short tandem repeat (STR) profiling every 6 months. Cells were cultured in F12, DMEM or RPMI‐1640 medium supplemented with 10% FBS in a humidified atmosphere of 5% CO_2_ at 37 °C.

### 2.2 Antibodies and reagents

Anti‐β‐tubulin (2148s), anti‐poly (ADP‐ribose) polymerase (PARP; 9542p), anti‐caspase3 (9662), anti‐cleaved caspase3 (9654), anti‐X‐linked inhibitor of apoptosis protein (XIAP; 14334), anti‐pEGFR (Tyr1068; 3777s), anti‐EGFR (4267s), anti‐p‐AKT (Ser473; 12694), anti‐p‐AKT (Thr308; 13038p), anti‐AKT (9272), anti‐p‐mTOR (Ser2448; 5536), anti‐mTOR (2983), anti‐p‐p70S6 (Thr389; 9234), anti‐p70S6 (2708), anti‐p‐S6 (Ser240/244; 5364), anti‐p‐S6 (Ser235/236; 2211), anti‐S6 ribosomal protein (2217) and anti‐LARP1 (70180) were purchased from Cell Signaling Technology (Danvers, MA, USA). Anti‐Bcl‐2 (ab32124), anti‐Bax (ab32503), anti‐Bcl‐XL (ab32370), anti‐Bim (ab32158), anti‐Bak (ab32371), anti‐Noxa (ab140129), anti‐Erk1 (pT202/pY204)+Erk2 (pT185 + pY187) (ab50011) and anti‐Erk1 + Erk2 (ab17942) were purchased from Abcam (Cambridge, MA, USA). Anti‐LARP1 (sc‐515873) was purchased from Santa Cruz biotechnology (Dallas, TX, USA). Goat anti‐rabbit IgG H+L, Alexa Fluor plus 647 (A32733) and goat anti‐mouse IgG H+L, Alexa Fluor plus 488 (A32723) were purchased from Invitrogen™, Thermo Fisher Scientific, Waltham, MA, USA. HRP goat anti‐mouse (A21010) and HRP goat‐anti‐rabbit (A21020) were purchased from Abbkine Scientific (Wuhan, Hubei, China).

### 2.3 Cell viability assay

Cell proliferation was measured by Cell Counting Kit‐8 (CCK‐8) assay (Bestbio, Shanghai, China). Cells were cultured in 96‐well plates at a concentration of 7 × 10^3^/well for 24 h. If necessary, cells were pretreated with 25 or 50 μm of Z‐VAD‐FMK for 4 h. Then cells were treated with indicated concentrations of compounds for 48 h. Supernatant was totally removed and 100 μL of CCK‐8 solution was added to each well and cultured for another 2 h at 37 °C. Cell viability was quantified using a SpectraMax M2e (Molecular Devices, San Jose, CA, USA) at 450 nm. Cell viability was calculated for each well as (absorbance 450 nm of treated cells/absorbance 450 nm of control cells) × 100%. Assays were performed on three independent experiments.

### 2.4 Apoptosis assay

Exponentially growing cells were seeded in 6‐well plates (2 × 10^5^/well) and cultured overnight in a 5% CO_2_ atmosphere at 37 °C. After treatment with Ibr‐7 for 24 h, cells were harvested and washed with PBS. Then cells were stained with Annexin V‐FITC Apoptosis Kit according to the manufacturer's instructions and analyzed by flow cytometry (Becton Dickinson, Franklin Lakes, NJ, USA). Assays were performed on three independent experiments.

### 2.5 Western blot analysis

After treated with different concentrations of compounds, total proteins were extracted using RIPA lysing buffer. A total of 40 μg of proteins were subjected to 12% SDS/PAGE and transferred to PVDF membrane (Bio‐Rad, Hercules, CA, USA). The membranes were blocked with 5% non‐fat milk at room temperature for 1 h, and then incubated with primary antibodies overnight at 4 °C. After washing with Tris buffered saline with Tween 20 (TBST), membranes were incubated with secondary antibodies at room temperature for another 1 h. The protein bands were visualized by adding ECL system WBKLS0050 (EMD Millipore, Billerica, MA, USA) and analyzed using Bio‐Rad Laboratories Quantity One software (Bio‐Rad).

### 2.6 Stable isotope labeling with amino acids in cell culture assay

A549 cells were cultured in F12 and supplemented with either (U‐12C6)‐l‐lysine (light) or (U‐13C6)‐l‐lysine (heavy) for at least eight generations. The heavy labeling efficiency was measured by mass spectrometer analysis. Cells were continuously maintained in SILAC medium until they reached the desired confluence. Cells were treated with 8 μm of Ibr‐7 for 8 h, and then harvested by trypsinization. The enriched fractions were analyzed by mass spectrometry.

### 2.7 Immunofluorescence

A549 cells were plated into Sigma Nunc® Lab‐Tek® II chambered coverglass 8 wells Sigma‐Aldrich (St. Louis, MO, USA) at 10 000 cells per chamber in complete medium and incubated for 24 h before use. The medium was replaced with 2, 4 or 8 μm of Ibr‐7 and cultured for 4 h. Cells were then rinsed with PBS twice before fixation in 4% formaldehyde for 20 min at room temperature. Cells were then rinsed with PBS three times and permeabilized by 0.2% Triton X‐100 for 10 min. After washing with PBS, cells were blocked by 5% BSA for 30 min and incubated with primary antibodies (phospho‐S6 ribosomal protein S235/236 and LARP1) overnight at 4 °C. Cells were washed in PBS and incubated with secondary antibody for 30 min. The slides were sealed with coverglasses using ProLong® Gold antifade reagent with DAPI (Invitrogen™, Thermo Fisher Scientific), and immediately observed by confocal microscope (Leica SP8, Wetzlar, Germany). The p‐S6 was visualized by excitation at 638 nm, and its fluorescence emission was observed using a 650–730 nm band‐pass filter. LARP1 was visualized by excitation at 488 nm and its fluorescence emission was observed using a 500–600 nm band‐pass filter. DAPI was excited at 405 nm and the emission was detected ranging from 420 to 480 nm.

### 2.8 Co‐immunoprecipitation assay

The co‐immunoprecipitation (Co‐IP) assay was performed according to a previously published protocol (Shen *et al*., 2018). Briefly, cells were lysed in a pre‐chilled lysis buffer supplemented with a protease inhibitor cocktail (Roche Applied Science, Mannheim, Germany). Protein A beads were incubated with anti‐ LARP1 or anti‐p‐S6(ser235, 236) for 4 h and then incubated with total protein lysates overnight. Western blot analysis of the precipitated protein was conducted as previously described in the section on western blot analysis.

### 2.9 Caspase‐3 activity assay

A549 cells (2 × 10^5^ cells/well, 6‐well plate) were incubated with 2 μm of Ibr‐7, 20 μm of ABT‐199 or the combination for 24 h. Cells were washed with PBS and lysed in cell lysis buffer. Caspase‐3 activity in cell lysates was determined colorimetrically using BioVision colorimetric caspase assay kits (Milpitas, CA, USA). Caspase‐3 employed chromophore conjugated peptides DEVD‐pNA and VEID‐pNA as substrates. Release of p‐nitroanilide (pNA) was assayed according to the supplier's instructions.

### 2.10 Clinical human tissue specimen

Clinical samples of lung cancer patients were obtained from Hangzhou First People's Hospital (Hangzhou, China). Written informed consent from patients and approval from the Institutional Research Ethics Committee of the hospital were obtained before the use of these clinical materials for research purposes.

### 2.11 Collagen gel droplet embedded 3D‐culture system

Collagen gel droplet embedded 3D‐culture system (CD‐DST) system was performed using a Tumor chemo‐sensitivity assay kit provided by Guangzhou Darui Biotechnology Co., Ltd (Guangzhou, China) (Hou *et al*., 2017). Briefly, 0.1–0.5 g freshly dissected human lung cancer tissues were digested by trypsin and incubated in collagen gel‐coated flasks. Then, cells were collected and incubated in a collagen gel droplet at the density of 4000 cells per droplet (the volume of each droplet was 30 μL). Cells were treated with 4 μm of ibrutinib, Ibr‐7 or AZD‐9291 for 24 h, and cultured for another 5 days at serum‐free medium. Cells were visualized by neutral red stain and observed using cell analysis system DR6690 (Guangzhou Darui Biotechnology Co., Ltd). Survival rates were calculated as (absorbance 540 nm of treatment group/absorbance 540 nm of control group) × 100%.

### 2.12 Sample collection and DNA extraction

Lung cancer tissues were formalin‐fixed and paraffin‐embedded (FFPE), and examined by pathological evaluation to ensure a tumor content of at least 20%. Tumor tissue DNA from FFPE was extracted using a FFPE DNA kit (Amoy Diagnostics Co., Ltd., Xiamen, China) according to manufacturer's instructions.

### 2.13 EGFR mutation detection

Epidermal growth factor receptor mutations of extracted DNA were identified using the ADx‐ARMS (amplification refractory mutation system) kit (Amoy Diagnostics Co., Ltd., Xiamen, China); all the experiments were performed according to the manufacturer's instructions (Cui *et al*., 2016).

### 2.14 Real‐time reverse transcription‐quantitative PCR

Total RNA was extracted from cells with TRIzol, precipitated with isopropyl alcohol, and rinsed with 70% ethanol. Single‐strand cDNA was prepared from the purified RNA using oligo (dT) priming (Invitrogen, Thermo Fisher Scientific), followed by SYBR‐Green (Qiagen, Hilden, Germany) and carried out using 7900HT Fast Real‐Time PCR system (Applied Biosystems Inc., Waltham, MA, USA). Assays were performed on three independent experiments. The following primers were used:

Mcl‐1, forward primer: 5′‐GGGCAGGATTGTGACTCTCATT‐3′,

reverse primer: 5′‐GATGCAGCTTTCTTGGTTTATGG‐3′;

GAPDH, forward primer: 5′‐GAGTCAACGGATTTGGTCGT‐3′,

reverse primer: 5′‐TTGATTTTGGAGGGATCTCG‐3′.

### 2.15 Tumor xenograft assay

All animal experiments were conducted according to the Institutional Animal Care and Use Committee (IACUC). A total of 5 × 10^6^ A549 cells was resuspended in 200 μL PBS and injected subcutaneously into each 4‐week‐old female nude mice. Once the tumor volume had reached 100–200 mm^3^, six mice were randomized into each group. Ibrutinib and Ibr‐7 were dissolved in 0.125 mL DMSO and vortexed for 10 min. Then, 2.375 mL of 20% HP‐beta‐cyclodextrin was added to the above mixture to make a final concentration of 6 mg·mL^−1^. Ibrutinib or Ibr‐7 was administrated orally twice per day at the dose of 60 mg·kg^−1^. Tumor volumes were determined from caliper measurements of tumor length (L) and width (W) according to the formula (L × W^2^)/2. The relative tumor volume (RTV) was calculated using the following formula: RTV = (tumor volume on measured day)/(tumor volume on day 0).

### 2.16 Statistical analysis

The results are expressed as the mean ± SD of at least three independent experiments. Differences between means were analyzed using Student's *t*‐test and were considered statistically significant when *P* < 0.05. Comparisons of more than two groups were evaluated by two‐way analysis of variance (ANOVA), and statistical significance was considered when *P* < 0.05. Statistical analyses and data visualization were performed using ibm spss version 22.0 (IBM SPSS, Inc., Chicago, IL, USA) and graphpad prism version 6.01 (GraphPad Software Inc., San Diego, CA, USA).

## 3 Results

### 3.1 Ibr‐7 induced apoptosis in NSCLC cells with EGFR wild‐type or mutated status

**Figure 1:**
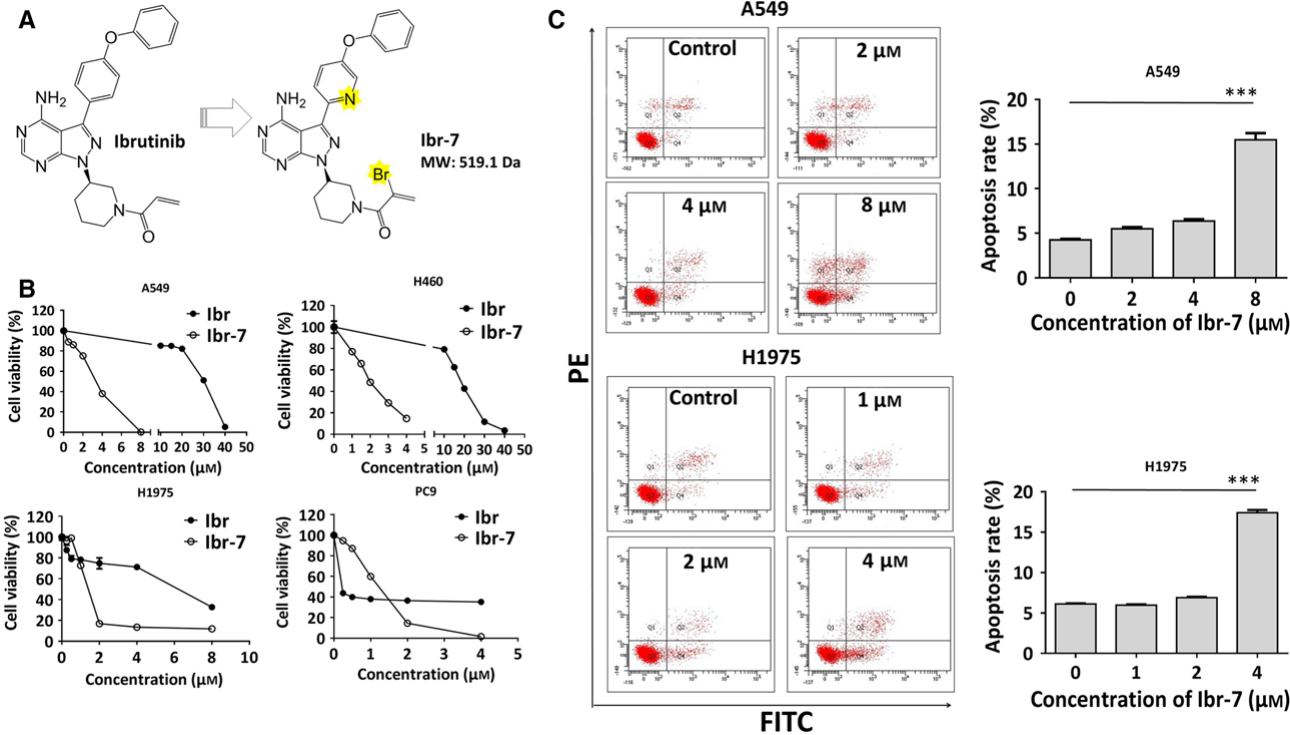
Ibr‐7 possesses potent anti‐proliferative activity against NSCLC cells by inducing apoptosis. (A) The chemical structure of Ibr‐7. (B) The dose‐dependent inhibitory effect of ibrutinib (Ibr) and Ibr‐7 on four non‐small lung cancer (NSCLC) A549, H460, H1975 and PC‐9 cell lines *in vitro*. Cells were treated with Ibr or Ibr‐7 for 48 h before CCK‐8 assay. (C) Ibr‐7 induced apoptosis in A549 and H1975 cells. Cells were treated with Ibr‐7 for 24 h before collection. Cells were then stained with Annexin V/PI and analyzed by flow cytometry. Three independent experiments were performed and data were presented as mean ± SD. ***P < 0.001.

Ibr‐7 is a newly synthesized derivative of ibrutinib that showed improved anti‐cancer activity against various cancer cells compared with its the parental compound (Fig. 1A, Table S1). The synthesis route and characterization of Ibr‐7 will be described in detail in our upcoming report. To determine the anti‐proliferation effects of Ibr‐7 in NSCLC cells, cells were incubated with ibrutinib or Ibr‐7 for 48 h before CCK‐8 assay. As expected, ibrutinib showed extreme sensitivity to PC‐9, which harbors EGFR 19 deletion mutation. The existence of EGFR T790M mutation in H1975 cells rendered a 100‐fold increase in the IC_50_ value compared with that of PC‐9 cells. In EGFR wild‐type A549 and H460 cells, the anti‐cancer activity of Ibr‐7 was obviously superior to that of ibrutinib (Fig. 1B), indicating different mechanisms of actions of Ibr‐7. In addition, Annexin V/PI stain was utilized to demonstrate the Ibr‐7 induced dose‐dependent apoptosis in A549 and H1975 cells after 24 h treatment (Fig. 1C). This apoptosis was further confirmed by DAPI stain, as evidenced by the appearance of apoptotic bodies with Ibr‐7 treatment for 24 h (Fig. S1).

**Figure 2:**
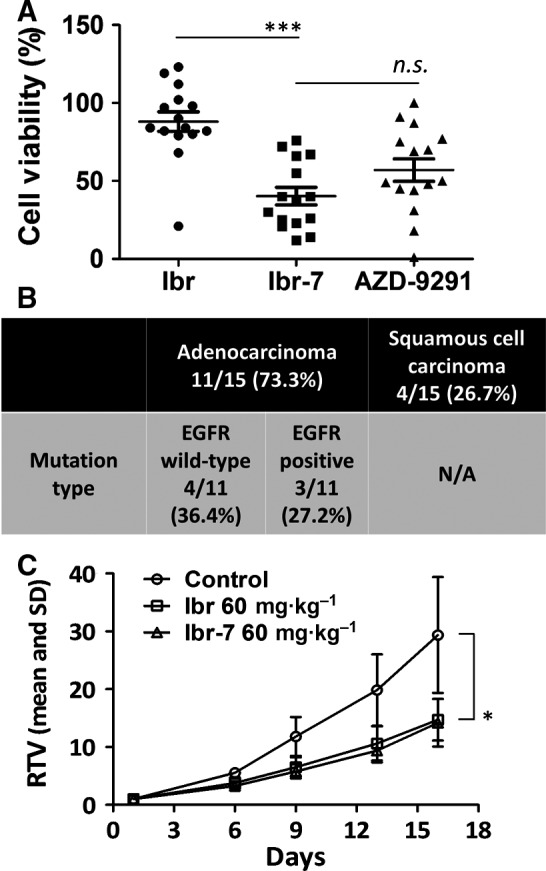
The anti‐tumor effect of Ibr‐7 in primary lung cancer cells and in xenograft nude mice. (A) Fifteen primary lung cancer cells were obtained and cultured using CD‐DST method. At treatment time, cells were treated with 4 μm of Ibr, Ibr‐7 or AZD‐9291 for 24 h. Treatment was then stopped and cells were cultured for another 5 days before analysis. (B) Pathological types of lung cancer were determined according to the pathology report for each patient. EGFR mutation was analyzed using amplification refractory mutation system (ARMS) detection. (C) A549 xenograft nude mice were administered 60 mg·kg^−1^ of ibrutinib or Ibr‐7 (six mice per group) every 2 or 3 days. Tumor volumes were determined according to the formula (L × W^2^)/2. The relative tumor volume (RTV) was calculated using the following formula: RTV = (tumor volume on measured day)/(tumor volume on day 0). Ibr, ibrutinib. Data were presented as mean ± SD. n.s., non‐significant, *P < 0.05, ***P < 0.001.

To demonstrate the anti‐proliferation activity of Ibr‐7, we collected a total of 15 primary lung cancer tissues and evaluated cell viability using a 3D culture model as described in Materials and methods. These primary lung cancer cells were treated with ibrutinib, Ibr‐7 or AZD‐9291 simultaneously, and the viable cell percentage was 88.1%, 40.3% and 57.0%, respectively (Fig. 2A) (Table S2). In addition, the EGFR mutation types were determined by ARMS‐PCR in seven of 15 samples; the other patients refused to take ARMS‐PCR because of the low mutation rate in lung squamous cancer. Nonetheless, these results demonstrated that Ibr‐7 showed a notable anti‐proliferative effect against lung cancer cells despite their EGFR mutation type *in vitro* (Fig. 2B).

We then used A549 xenograft nude mice model to evaluate the *in vivo* anti‐tumor effect of Ibr‐7 and ibrutinib. As shown in Fig. 2C, by calculating the relative tumor volume (RTV) at the dose of 60 mg·kg^−1^ via intragastric administration twice per day, Ibr‐7 displayed the same anti‐tumor activity as ibrutinib, without affecting the mice bodyweight (Fig. S2). By studying the pharmacokinetics of ibrutinib and Ibr‐7, we found that the C_max_ of Ibr‐7 ibrutinib was 304 ng·mL^−1^ (Table S3), nearly half the value of ibrutinib (data not shown). Therefore, the bioavailability of Ibr‐7 needs to be improved for further applications, through either molecular modification or biomaterial encapsulation.

### 3.2 Ibr‐7 suppressed AKT/mTOR/S6 phosphorylation

**Figure 3:**
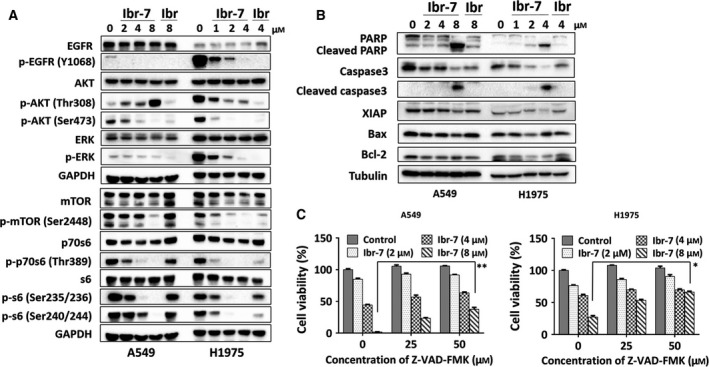
Ibr‐7 induced caspase‐dependent apoptosis in NSCLC by suppressing mTORC1/S6 pathway. (A) Ibr‐7 suppressed phosphorylated proteins in the Akt/mTOR pathway. A549 and H1975 cells were treated with indicated concentrations for 8 h before western blotting analysis. (B) Cells were treated with ibrutinib (Ibr) or Ibr‐7 for 24 h before western blotting assay. (C) Cells were pretreated with indicated concentrations of Z‐VAD‐FMK for 4 h and then cultured with various concentrations of Ibr‐7 for another 48 h before CCK‐8 cell viability assay. Three independent experiments were performed and data were presented as mean ± SD. *P < 0.05, **P < 0.01.

ELISA was used to determine the inhibitory effect of Ibr‐7 on five kinases after molecular modification. Both Ibr‐7 and ibrutinib showed high selectivity in EGFR, the IC_50_ value was 61 and 2.3 nm, respectively (Table S4). Using western blotting assay, we found that both Ibr‐7 and ibrutinib could intensely downregulate the level of p‐EGFR after 2 h treatment (Fig. S3). In addition, ibrutinib and Ibr‐7 slightly inhibited the phosphorylation of ErbB‐2 and ErbB‐4 after in A549 cells (Fig. S4), which was consistent with previously published results (Grabinski and Ewald, 2014). While observing the downstream phosphorylation status of p‐mTOR, p‐p70S6 and p‐S6, a pronounced difference occurred at a concentration of 8 and 4 μm for A549 and H1975 cells, respectively, between ibrutinib and Ibr‐7 (Figs 3A and S5). Ibr‐7 potently downregulated p‐mTOR, p‐p70S6 and p‐S6 in a dose‐dependent manner, and this effect was further confirmed by SILAC assay (Table 1). Since p‐S6 is the downstream functional factor that controls the translational process, we attempted to determine the role of p‐S6 in the Ibr‐7 antitumor effect. Transfection of active p‐S6 plasmid partially elevated the level of p‐S6 (240/244) with Ibr‐7 treatment, without affecting the basal p‐S6 level (Fig. S6). Consistently, cell viability increased slightly after transfection with p‐S6 plasmid, suggesting the co‐participation of alternative factors in controlling translation processes.

**Table 1:** Changes of phosphorylated proteins determined by SILAC assay after treatment with Ibr‐7 in A549 cells. Labeled A549 cells were treated with 8 μm of Ibr‐7 for 8 h before mass spectrometry analysis. Decrease fold refers to changes in phosphorylated protein of treated cells versus untreated cells

Protein accession number	Protein name	Position	Decrease fold	*P* value
P00533	EGFR	991	2.03	0.001
P00533	EGFR	693	1.76	0.001
P42345	mTOR	2481	2.23	0.004
Q15418	RPS6KA1	359	1.59	0.002
Q15418	RPS6KA1	363	1.54	0.001
P62753	RPS6	244	2.17	0.022
P20042	EIF2S2	67	1.99	0.002
P20042	EIF2S2	111	1.58	0.008
Q6PKG0	LARP1	766	4.70	0.000
Q6PKG0	LARP1	824	2.34	0.000
Q6PKG0	LARP1	548	1.90	0.005

Since it remains questionable that whether EGFR plays an essential role in the Ibr‐7 anti‐tumor effect in lung cancer cells, we knocked down the expression of EGFR by siRNA transfection in A549 cells, and determined the cell proliferation rate by CCK‐8 assay (Fig. S7A). There was no significant difference between negative control cells and those transfected with siEGFR after exposure to either Ibr‐7 or ibrutinib (Fig. S7B). Additionally, by analyzing downstream proteins including mTOR and S6, siEGFR showed only a negligible effect on the phosphorylation of these proteins, except for p‐S6 (Ser235/236) (Fig. S7C). Although EGFR was crucial to the proliferation of lung cancer cells, our results suggested that EGFR was not an important target of Ibr‐7 in A549 cells, even considering the intense inhibitory effect of Ibr‐7 on phosphor‐EGFR.

When prolonging the treatment time to 24 h, mitochondrion‐mediated apoptotic proteins were measured; these cells tended to undergo apoptosis (Fig. 3B). Additionally, Ibr‐7 induced apoptosis could be reversed by pretreatment with pan‐caspase inhibitor, suggesting the crucial role of caspases 3/7 in Ibr‐7 in causing apoptosis (Fig. 3C).

### 3.3 Ibr‐7 impeded the protein synthesis of Mcl‐1 via disruption p‐S6/LARP1

**Figure 4:**
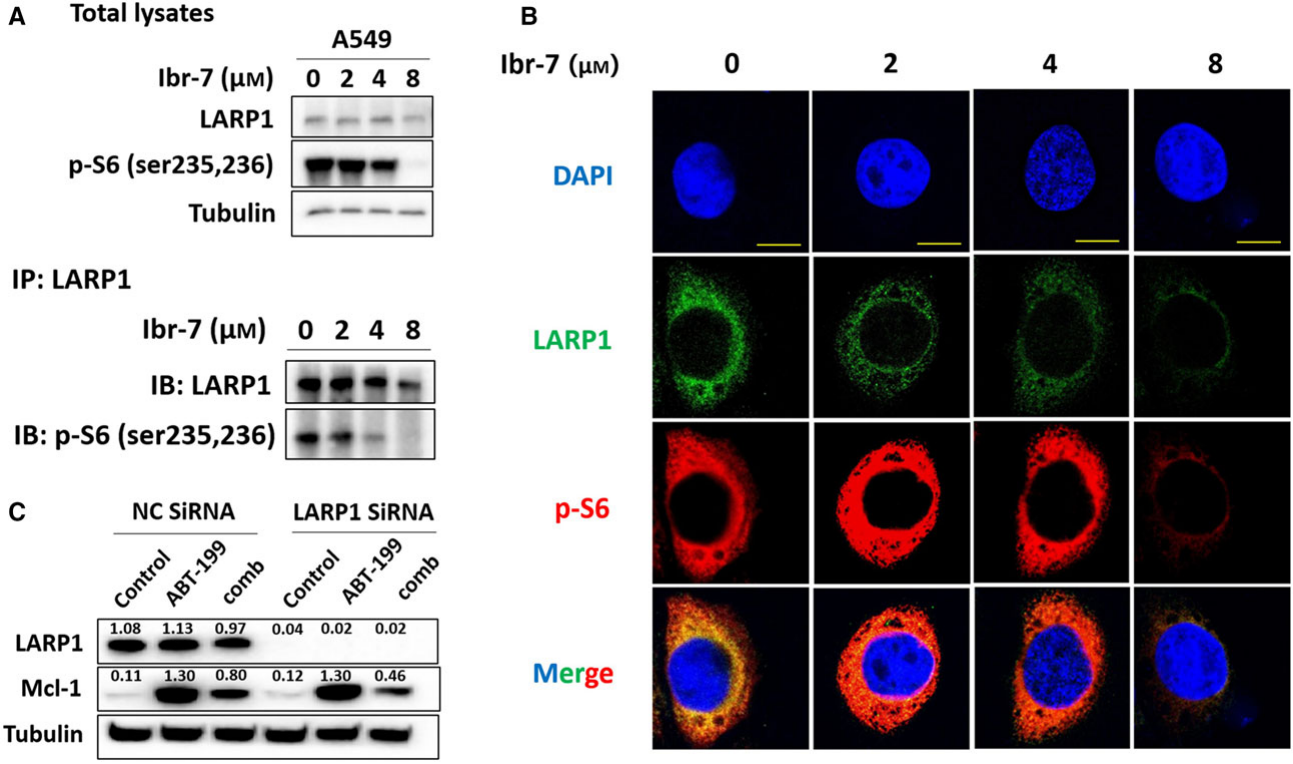
Ibr‐7 impaired protein interactions between LARP1 and p‐S6. (A) A549 cells were treated with Ibr‐7 for 8 h before the co‐immunoprecipitation assay. (B) After treatment with Ibr‐7 for 8 h, cells were fixed and incubated with primary and secondary antibody, followed by confocal microscopy. (C) The expression of LARP1 was eliminated by LARP1 siRNA. A549 cells were treated with ABT‐199 or a combination of ABT‐199 and Ibr‐7 for 8 h before western blotting analysis. Scale bar: 10 μm.

In addition to the inhibition of mTORC1/S6, Ibr‐7 showed a pronounced inhibitory effect on LARP1. Since LARP1 was reported to function downstream of mTOR, we assumed that LARP1 might co‐participate with p‐S6 in Ibr‐7‐caused protein synthesis suppression. Co‐immunoprecipitation was used to demonstrate the interaction between p‐S6 and LARP1, and the presence of Ibr‐7 could impede the co‐localization of p‐S6 and LARP1 in a dose‐dependent manner (Fig. 4A). We then used confocal microscopy to study the subcellular localization of p‐S6 and LARP1. As shown in Fig. 4B, most LARP1 co‐localized with p‐S6 at cytoplasm, and treatment with Ibr‐7 clearly impaired the interaction between LARP1 and p‐S6. In contrast, silencing LARP1 had an insignificant influence on the cytotoxicity of Ibr‐7, suggesting that LARP1 might not be the direct target of Ibr‐7 (Fig. S8).

Mcl‐1 is a critical anti‐apoptotic protein belonging to the Bcl‐2 family. The downregulation of Mcl‐1 would therefore sensitize a series of Bcl‐2 inhibitors, such as ABT‐199. In our study, silencing Mcl‐1 significantly enhanced the anti‐proliferative activity of ABT‐199 mono‐treatment as well as combined treatment of ABT‐199 and Ibr‐7 (Fig. S9). In A549 cells, treatment with ABT‐199 alone enhanced the expression level of Mcl‐1, whereas combinatorial treatment with ABT‐199 and Ibr‐7 reduced the Mcl‐1 level by 40% (the band intensity dropped from 1.30 to 0.80). Knockdown LARP1 by siRNA almost eliminated LARP1 expression, but rendered a moderate decline in Mcl‐1 level after combination treatment. These results suggested that LARP1 might not fully function downstream of p‐S6 but partially co‐participates with p‐S6 in the protein synthesis of Mcl‐1 (Fig. 4C).

**Figure 5:**
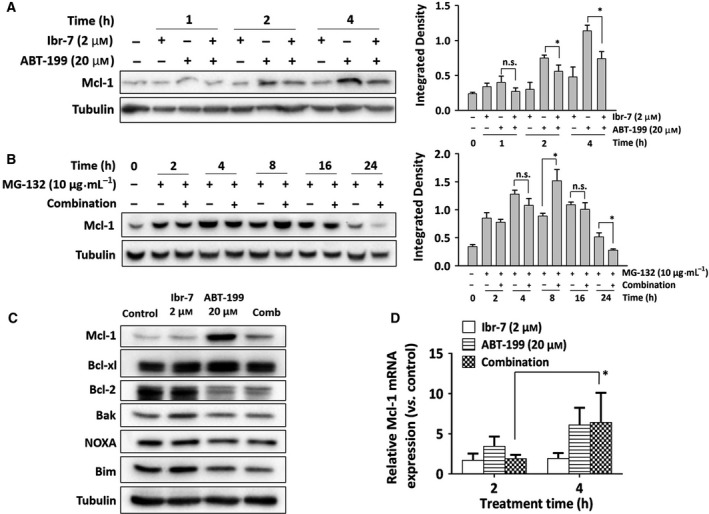
Ibr‐7 could overcome ABT‐199 triggered Mcl‐1 up‐regulation via inhibition of the protein synthesis pathway. (A) A549 cells were treated with Ibr‐7 or A549 for indicated times before western blotting assay. (B) A549 cells were co‐incubated with MG‐132 and a combination treatment of ABT‐199 (20 μm) and Ibr‐7 (2 μm) for different times before western blotting assay. (C) A549 cells were treated with Ibr‐7, ABT‐199 or a combination for 2 or 4 h. Total RNA was extracted from A549 cells to undergo RT‐qPCR assay. (D) A549 cells were treated with Ibr‐7, ABT‐199 or a combination for 24 h before western blotting assay. Three independent experiments were performed, and data were presented as mean ± SD. n.s., non‐significant, *P < 0.05.

To confirm the inhibitory effect of Ibr‐7 on Mcl‐1, we used western blotting to analyze Mcl‐1 protein level at different treatment times. At 2 h treatment, ABT‐199 alone was able to increase Mcl‐1, whereas co‐treatment with Ibr‐7 successfully reversed the elevated Mcl‐1 level (Fig. 5A). Pretreatment with MG‐132, which is a proteasome inhibitor, accelerated Mcl‐1 protein degradation after combinatorial treatment (Fig. 5B); however, 10 μm of MG‐132 had no cytotoxicity effect on A549 cells (Fig. S10). Pretreatment with cycloheximide (CHX) did not influence the degradation of Mcl‐1 (Fig. S11). Additionally, we analyzed the mRNA level of Mcl‐1 after mono‐ or co‐treatment with Ibr‐7 and ABT‐199; neither Ibr‐7 or combination treatment influenced the transcription level of Mcl‐1 (Fig. 5C). As BH‐3‐only proteins were reported to bind with Mcl‐1, causing Mcl‐1 degradation, we determine the protein level of Bak, NOXA and Bim and found no obvious up‐regulation of these three proteins (Fig. 5D). Therefore, these results indicated that Ibr‐7 could overcome the elevated Mcl‐1 induced by ABT‐199 mono‐treatment via protein synthesis inhibition but not by proteasomal degradation.

### 3.4 Synergistic effect of Ibr‐7 combined with ABT‐199 against NSCLC cells

**Figure 6:**
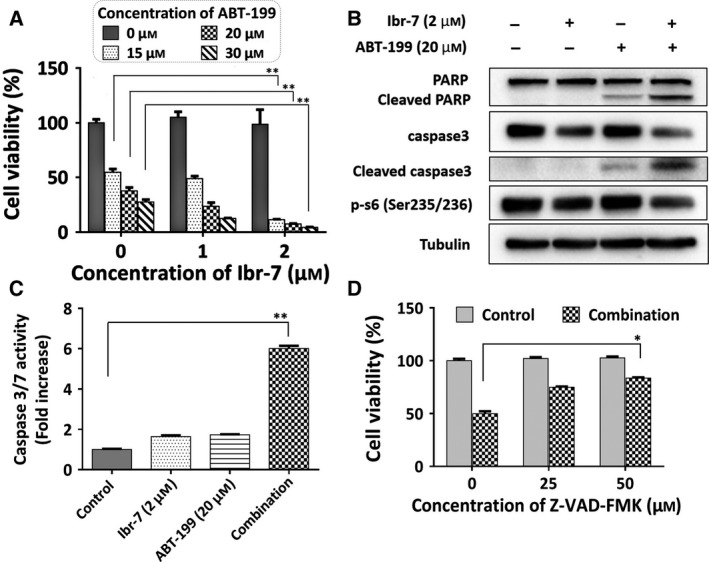
Ibr‐7 synergized with ABT‐199 in NSCLC A549 cells by triggering apoptosis. (A) A549 cells were treated with Ibr‐7, ABT‐199 or a combination for 48 h before cell viability assay using Cell Counting Kit‐8 (CCK‐8). (B) A549 cells were treated with Ibr‐7, ABT‐199 or a combination for 24 h before western blotting assay. (C) After treatment with Ibr‐7, ABT‐199 or a combination for 24 h, cells were lysed and underwent caspase activity analysis using BioVision colorimetric caspase assay kits. (D) Cells were pretreated with Z‐VAD‐FMK for 4 h and then cultured with a combination of Ibr‐7 and ABT‐199 for 48 h before CCK‐8 cell viability assay. Three independent experiments were performed and data were presented as mean ± SD. *P < 0.05, **P < 0.01.

Single treatment of ABT‐199 was ineffective in killing A549 and H1975 cells. The calculated IC_50_ values were larger than 10 μm in these two lung cancer cell lines (Fig. S12). As Ibr‐7 was capable of overcoming the Mcl‐1 caused by ABT‐199 mono‐treatment, it was assumed that Ibr‐7 would sensitize NSCLC cells to ABT‐199. As shown in Fig. 6A and Table 2, Ibr‐7 combined with ABT‐199 had strong synergistic effects in killing A549 cells; the combination index was 0.25 at optimal concentrations. Meanwhile, the role of caspases was determined by western blotting and caspase 3/7 activity assay. The activity of caspases was apparently elevated after combination treatment (Fig. 6B,C). Moreover, addition of Z‐VAD‐FMK could greatly reverse the apoptosis caused by combination treatment (Fig. 6D).

**Table 2:** Combination index (CI) value of combinatorial effect on A549 cells. CI value was calculated using calcusyn software (Biosoft v.2.1, Cambridge, UK) based on the anti‐proliferation data obtained from Fig. 6A. 0.9 ≤ CI≤1.1 represents additive effect (grey shade); 0.4 ≤ CI<0.9 represents moderate synergism (green shade); CI<0.4 represents strong synergism (yellow shade)

CI value		Ibr‐7 (μm)	
		1	2
ABT‐199 (μm)	15	0.97	0.26
	20	0.57	0.25
	30	0.57	0.25

## 4 Discussion

Although ibrutinib has shown its extreme sensitivity towards chronic lymphocytic leukemia by targeting BTK, the application of ibrutinib in treating solid tumors has been fraught with obstacles (Byrd *et al*., 2015, 2013). Due to the structural similarity of BTK, EGFR and bone marrow X kinase (BMX), ibrutinib could potently inhibit BMX and EGFR in NSCLC cells, but only those with an EGFR‐mutant (Ahn *et al*., 2017; He *et al*., 2017; Molina‐Cerrillo *et al*., 2017; Wang *et al*., 2016; Wu *et al*., 2015). To enhance the anti‐cancer activity of ibrutinib in lung cancer cells, we synthesized a series of derivatives and obtained Ibr‐7 as the most promising candidate agent (Table S1). In this study, we focused on lung cancer. The antitumor activity of Ibr‐7 and ibrutinib was evaluated on four lung cancer cell lines with varying genetic backgrounds. PC‐9 harbors EGFR exon 19 deletion, which is an EGFR TKI‐sensitive mutation. H1975 has both an EGFR‐sensitive mutation (exon 21 L858R) and a resistance mutation (T790M) (Ichihara *et al*., 2017). Both A549 and H460 cells are EGFR wild‐type cell lines. The IC_50_ values of Ibr‐7 against these four lung cancer cell lines were about 1–4 μm (Table S1). We further validated the anti‐cancer activity of Ibr‐7 on primary lung cancer cells using CD‐DST assay. At a concentration of 4 μm, Ibr‐7 exhibited a stronger anti‐proliferation effect than ibrutinib or even AZD9291 in the 3D‐cultured model.

To explore the underlying mechanisms responsible for superior anti‐cancer effects of Ibr‐7, we chose A549 and H1975 cell lines for the following experiments. A kinase substrate screening assay was applied to determine the direct substrate of ibrutinib and Ibr‐7 (Table S4). Both ibrutinib and Ibr‐7 showed a direct inhibitory effect on EGFR with IC_50_ values of 2.3 and 61.0 nm, respectively; the IC_50_ values of PI3Ka, mTOR, AKT1 and p70S6K were larger than 1000 nm. In Fig. 3A, both ibrutinib and Ibr‐7 potently downregulated EGFR^Y1068^_,_ analyzed by western blotting. While ibrutinib was capable of decreasing the phosphorylation of AKT^T308/S473^ and ERK, Ibr‐7 dramatically suppressed the phosphorylation of downstream signaling, including mTOR^S2448^, p‐p70S6 (also known as p‐S6K) and p‐S6. Since the phosphorylation of AKT^S473^ was mainly regulated by mTOR^S2481^, it appeared that both ibrutinib and Ibr‐7 could influence mTORC2; however, only Ibr‐7 strongly de‐phosphorylated mTORC1 (Copp *et al*., 2009; Gao *et al*., 2018). Therefore, we assumed that Ibr‐7 modulated protein synthesis to exhibit its antitumor effect towards NSCLC cells by inhibiting the mTORC1/pS6 pathway (Ma and Blenis, 2009).

Using SILAC assay to screen the phosphorylation changes in total proteins after Ibr‐7 treatment, we obtained results consistent to western blotting. Interestingly, we found that LARP1 was significantly affected by Ibr‐7 treatment. LARP1 was recently found to control the translation of terminal oligopyrimidine motif (TOP mRNA), and this process was precisely regulated by mTORC1 (Mura *et al*., 2015). Inhibition of mTOR signaling by rapamycin could severely impede the function of LARP1 (Fonseca *et al*., 2015). TOP mRNA was reported to be regulated by phosphorylation of S6, which could control the binding affinity of TOP mRNA and ribosome via phosphorylation (Hornstein *et al*., 2001; Jefferies *et al*., 1994). These studies encouraged us to study the relation between p‐S6 and LARP1. We found direct protein interactions between p‐S6 and LARP1, which could be substantially impaired by Ibr‐7 treatment. In addition, silencing of LARP1 only partially affected the protein level of Mcl‐1 under Ibr‐7 treatment, illustrating that LARP1 might not function downstream of p‐S6 but co‐activate the translation of ribosomal translation components.

In previous studies, Mcl‐1 was determined to be a critical anti‐apoptotic factor controlling drug resistance in solid tumors, especially resistance to Bcl‐2 inhibitors (Li *et al*., 2018; Souers *et al*., 2013; Zhang *et al*., 2016). The down‐regulation of Mcl‐1 could significantly enhance the antitumor effect of Bcl‐2 inhibitors (Butterworth *et al*., 2016; Teh *et al*., 2018; Tong *et al*., 2017). In the Bcl‐2 family, Bim, Puma and Noxa were reported to bind directly to Mcl‐1 for proteasomal degradation (Chen *et al*., 2005; Delbridge *et al*., 2016). In our study, Noxa and Bim were found unchanged after combination treatment of ABT‐199 and Ibr‐7. Additionally, when cells were pretreated with MG‐132, combination treatment accelerated Mcl‐1 degradation, suggesting that Mcl‐1 was manipulated in the process of protein synthesis. Therefore, Ibr‐7 could reverse the elevated Mcl‐1 protein expression caused by ABT‐199 single treatment, showing a great synergistic effect with ABT‐199 in A549 cells.

## 5 Conclusions

In this study, we replaced acrylamide and diphenyl ether to generate an ibrutinib derivative, Ibr‐7, which showed superior cytotoxicity to ibrutinib against solid tumors. Ibr‐7 dramatically suppressed the phosphorylation of EGFR and mTORC1/S6 signaling, thus exerting its potent anti‐cancer activity against NSCLC cells. To take advantage of its unique mechanism of action, Ibr‐7 could be utilized to sensitize ABT‐199 by impairing the protein synthesis of Mcl‐1. Therefore, this study not only suggested an effective strategy to improve the anti‐cancer activity of BTK inhibitors against solid tumors, but also provided meaningful insights into the design and development of kinase inhibitory agents.

## Conflict of interest

The authors declare no conflict of interest.

## Author contributions

BZ and NML designed the experiments, analyzed data and wrote the manuscript. LLW performed immunoblotting experiments, QZ performed qRT‐PCR and *in vivo* antitumor experiments, HJ and RLH dissected lung cancer tissues and performed 3D culture, XLZ and XGL provided the compounds, YYY performed the immunofluorescence experiments.

## Supporting information


**Table S1.** The IC_50_ values of ibrutinib and Ibr‐7 against various cancer cell lines.
**Table S2.** Cell viability of 15 primary lung cancer cells after treatment with 4 μm of compounds for 24 h, and cultured for another 120 h before neutral red stain and fixation.
**Table S3.** PK parameters of Ibr‐7 in SD rat (intragastric 30 mg·kg^−1^).
**Table S4.** The inhibitory activity of ibrutinib and Ibr‐7 on five kinases.
**Fig. S1.** DAPI stain of cell nucleus.
**Fig. S2.** Bodyweights of xenograft nude mice after administration of Ibr (ibrutinib) or Ibr‐7, 60 mg·kg^−1^ twice a day.
**Fig. S3.** Western blotting assay of EGFR and p‐EGFR.
**Fig. S4.** Western blotting assay of p‐ErbB‐2, ErbB‐2, p‐ErbB‐4 and ErbB‐4.
**Fig. S5.** Quantitative analysis of proteins.
**Fig. S6.** Active p‐S6 overexpression slightly affects the anti‐proliferation effect of Ibr‐7.
**Fig. S7.** Knockdown of EGFR had negligible effects on the anti‐proliferation effect of Ibr‐7.
**Fig. S8.** Knockdown of LARP1 did not undermine the anti‐proliferation effect of Ibr‐7.
**Fig. S9.** Mcl‐1 played a key role in the antitumor effect of ABT‐199 and combination treatment.
**Fig. S10.** MG‐132 showed no cytotoxicity in A549 cells.
**Fig. S11.** CHX did not expedite the degradation of Mcl‐1.
**Fig. S12.** The cytotoxicity of ABT‐199 on A549 and H1975 cells.
