# Supplementary material for: The Ibr‐7 derivative of ibrutinib exhibits enhanced cytotoxicity against non‐small cell lung cancer cells via targeting of mTORC1/S6 signaling
Source: Mol Oncol. 2019 Feb 22;13(4):946–58. doi: 10.1002/1878-0261.12454 (PMC6441926; doi:10.1002/1878-0261.12454)

**Supplementary Data**

**The Ibr-7 derivative of Ibrutinib exhibits enhanced cytotoxicity against non-small cell lung cancer (NSCLC) cells via targeting of mTORC1/S6 signaling**

Bo Zhang^1^*, Linling Wang^1,2,6^*, Qi Zhang^1^*, Youyou Yan^1^, Hong Jiang^3^, Runlei Hu^3^, Xinglu Zhou^4^, Xingguo Liu^4^, Jianguo Feng^5^, Nengming Lin^1,2^#

^1^Translational medicine research center, Affiliated Hangzhou First People’s Hospital, Zhejiang University School of Medicine, Hangzhou, Zhejiang, China

^2^Affiliated Hangzhou First People’s Hospital, Zhejiang Chinese Medical University, Hangzhou, Zhejiang, China

^3^Department of Thoracic Surgery, Affiliated Hangzhou First People’s Hospital, Zhejiang University School of Medicine, Hangzhou, Zhejiang, China

^4^Hangzhou Hertz Pharmaceutical Co., Hangzhou, Zhejiang, China

^5^Cancer Research Institute, Zhejiang Cancer Hospital, Hangzhou, Zhejiang, China

^6^Shaoxing Hospital of Traditional Chinese Medicine, Shaoxing, Zhejiang, China

* These authors contributed equally to this work.

**Corresponding Author:** Nengming Lin, Translational medicine research center, Affiliated Hangzhou First People’s Hospital, Zhejiang University School of Medicine, Hangzhou, 310006 Zhejiang, China.

Phone: 86-571-56007905; Fax: 86-571-87914773

E-mail: lnm1013@163.com

Table S1. The IC50 values of Ibrutinib and Ibr-7 against various cancer cell lines.


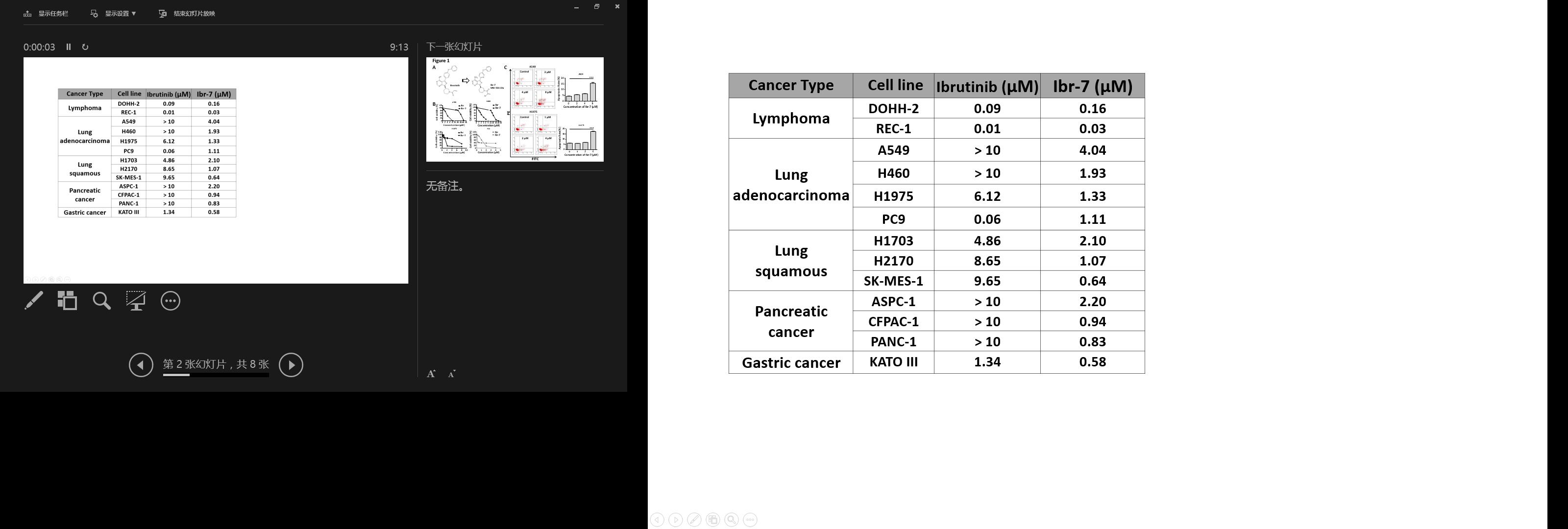


Table S2. Cell viability of 15 primary lung cancer cells after treatment with 4 μM of compounds for 24 h, and cultured for another 120 h before neutral red stain and fixation. Cell viability was calculated observed using specific cell analysis system DR6690 (Guangzhou Darui Biotechnology Co., Ltd, Guangzhou, China). Survival rates were calculated as (O.D. 540nm treatment group/O.D. 540nm control group) × 100%.


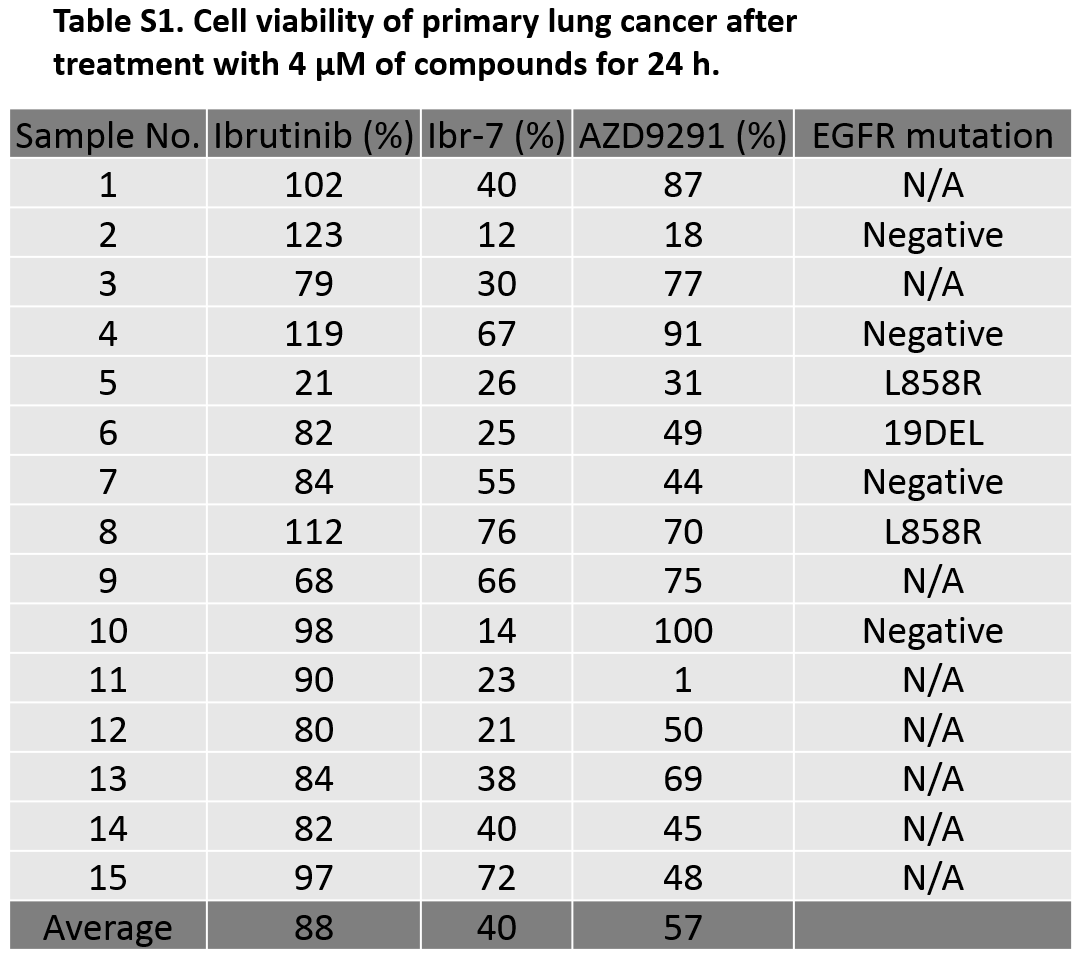


Table S3. PK parameters of Ibr-7 in SD rat (i.g 30 mg/kg). Ibr-7 was orally administrated at the dose of 30 mg/kg. At each time point, 0.1 ml of rat blood was gained through retro-orbital plexus and then centrifuged at 5,000 rpm, 4 ℃ for 10 min. 10 μl of sample was mixed with 100 μl of internal reference (Buspirone 50 ng/ml, terfenadine 50 ng/ml), and vortexed for 60 s. After centrifuging at 12,000 rpm for 3 min, the supernatant was collected and transferred to an equal volume of distilled water into the 96-well plate. The final injection volume was 10 μl. Samples were analyzed by LC-MS/MS (API 4000QTRAP) and data was processed by Analyst v 1.5.1.


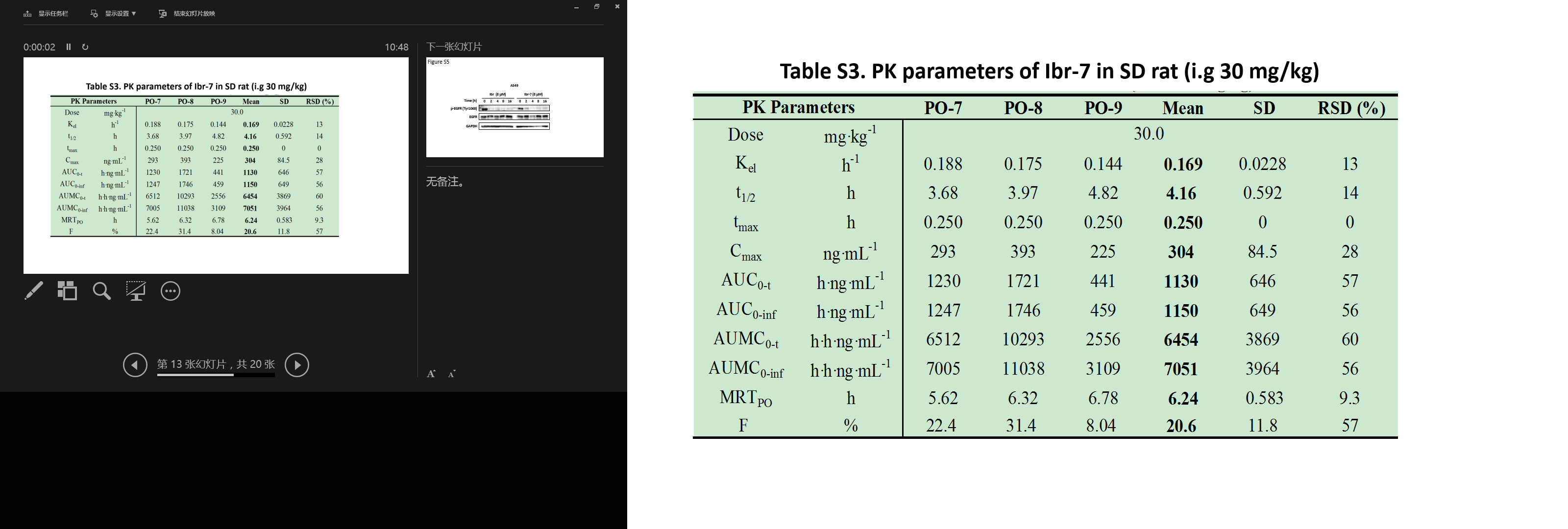


Table S4. The inhibitory activity of Ibrutinib and Ibr-7 on five kinases.

| **IC50 (nM)** | **p70S6K** | **AKT1** | **EGFR** | **PI3Ka** | **mTOR** |
| --- | --- | --- | --- | --- | --- |
| **Ibrutinib** | >1000 | >1000 | 2.3 | >1000 | >1000 |
| **Ibr-7** | >1000 | >1000 | 61 | >1000 | >1000 |
| **Internal**  **Reference** | 0.49 | 7.1 | 36 | 9.1 | 4.5 |

Figure S1. DAPI stain of cell nucleus. Cells were treated with indicated compounds for 24 h before fixation and stained with DAPI stain. Fluorescence was observed under microscopy (Nikon Eclipse Ti). Scale bars: 20 μm. Magnification: 400 X.


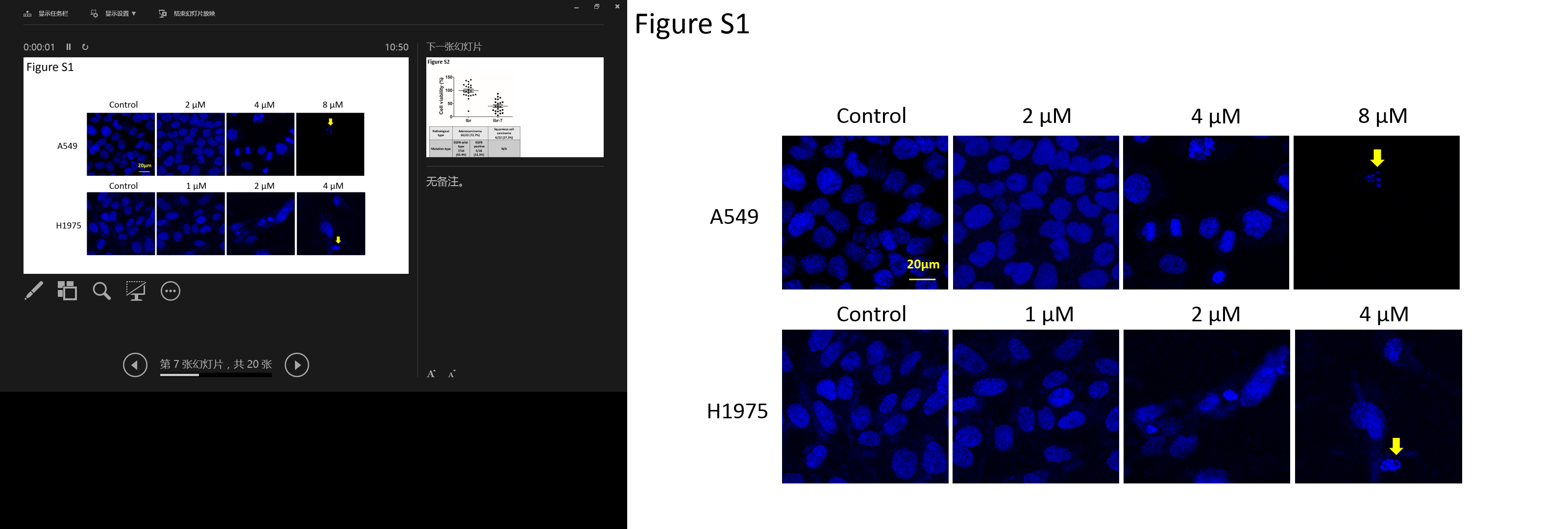


Figure S2. Body weights of xenograft nude mice after administrated with Ibr (Ibrutinib) or Ibr-7 at the dose of 60 mg/kg b.i.d.. Data were presented as mean ± SD.

Figure S3. Western blotting assay of EGFR and p-EGFR. A549 cells were treated with Ibr (Ibrutinib) or Ibr-7 at the concentration of 8 μM for indicated times.


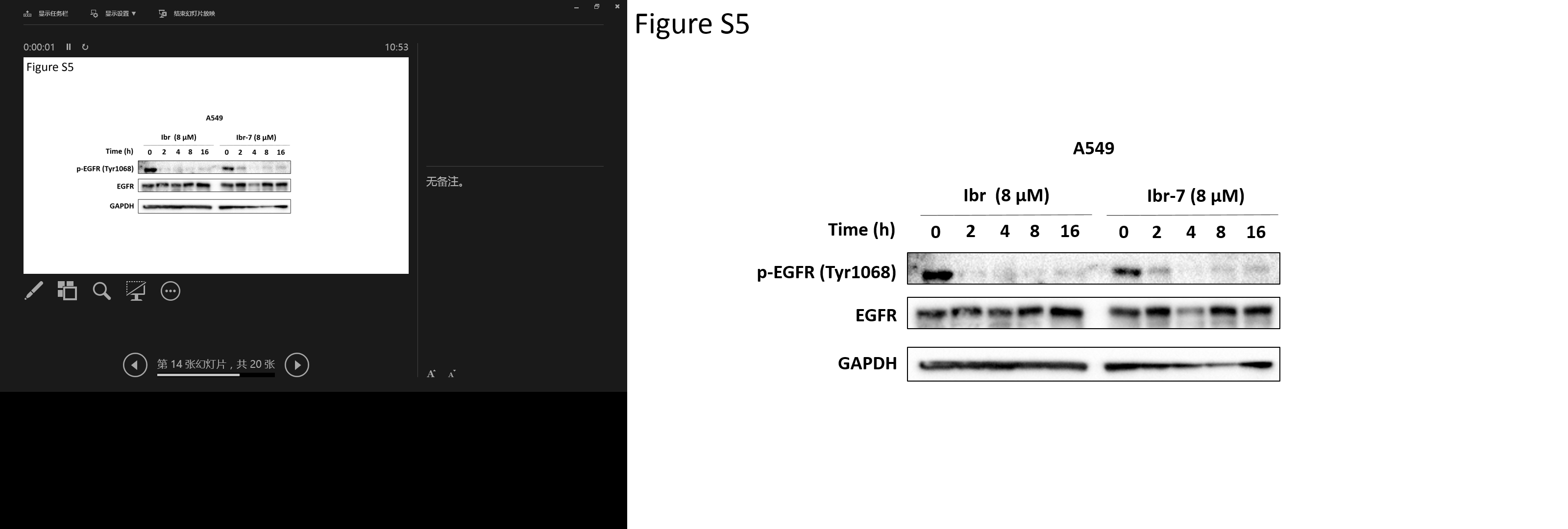


Figure S4. Western blotting assay of p-ErbB-2, ErbB-2, p-ErbB-4 and ErbB-4. A549 cells were treated with Ibr (Ibrutinib) or Ibr-7 at the concentration of 8 μM for indicated times.


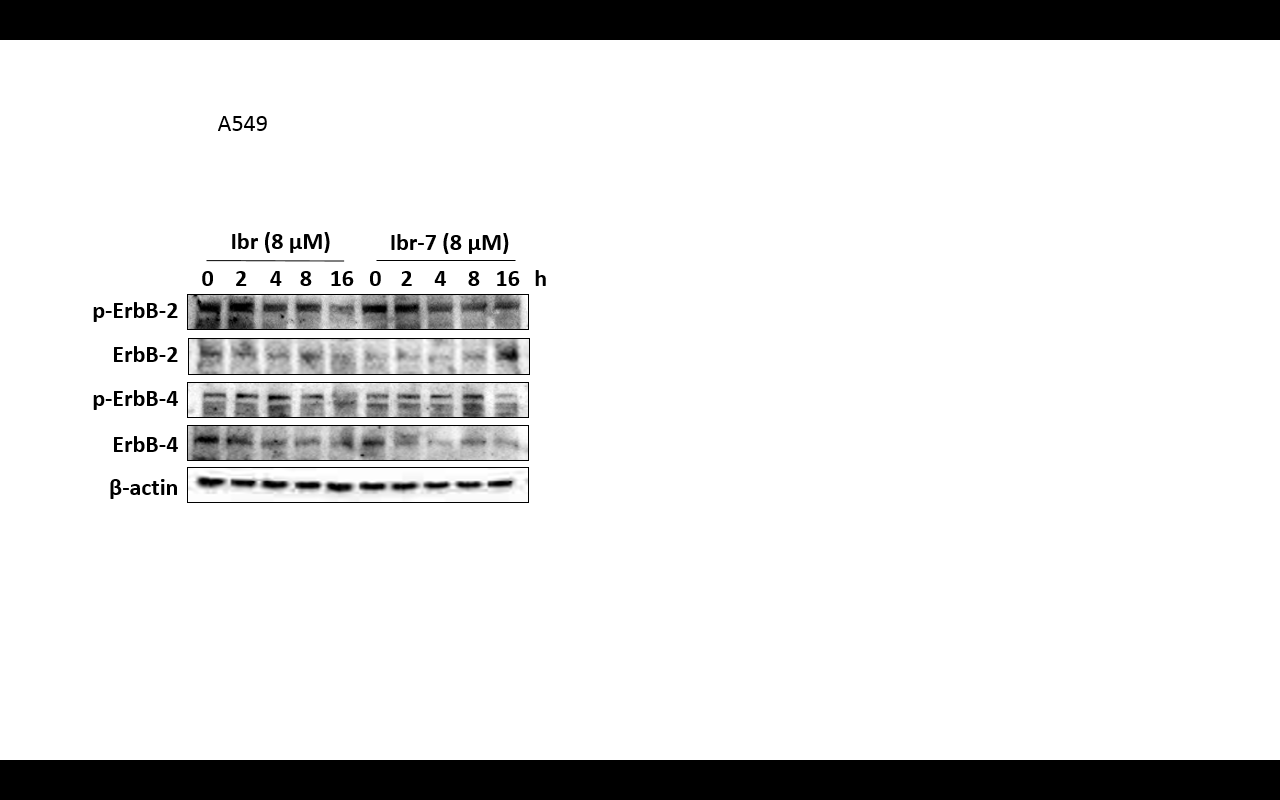


Figure S5. Quantitative analysis of proteins. The integrated density of p-Akt, p-ERK, p-mTOR, p-p70S6 and p-S6 in Fig 3A were quantitatively analyzed using Image J (1.49 v). Statistical analysis was conducted according to three independent experiments. Three independent experiments were performed, and data were presented as mean ± SD. *** p < 0.001, ** p < 0.01, * p < 0.05, n.s. = non-significant.


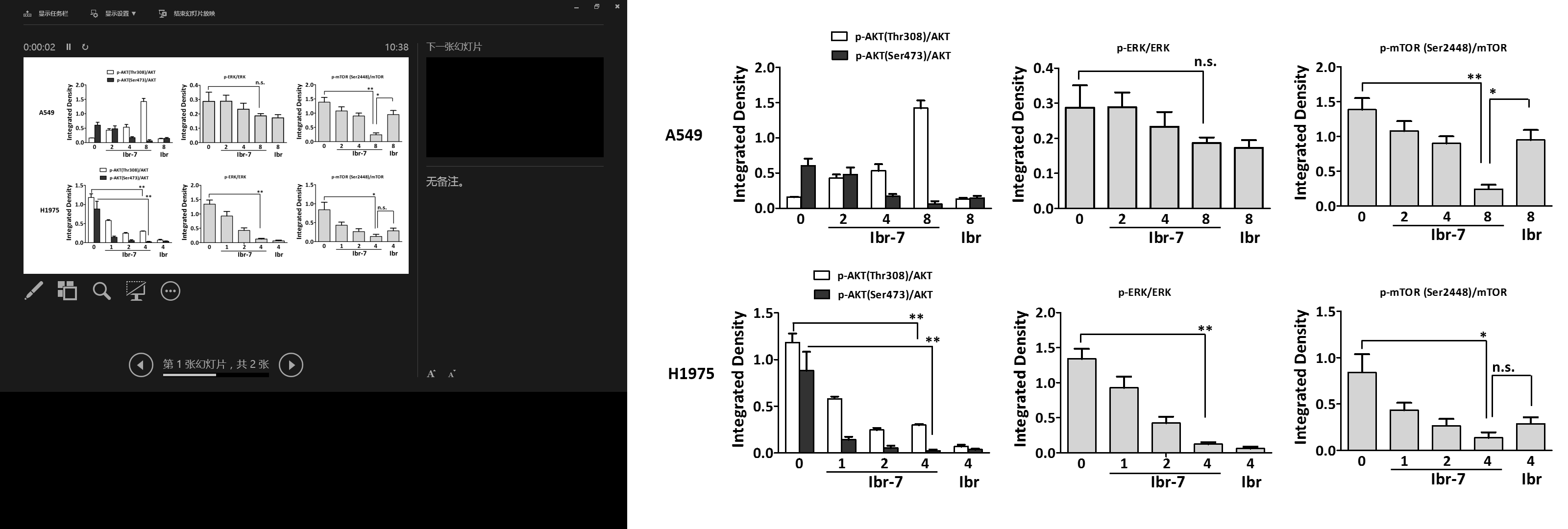


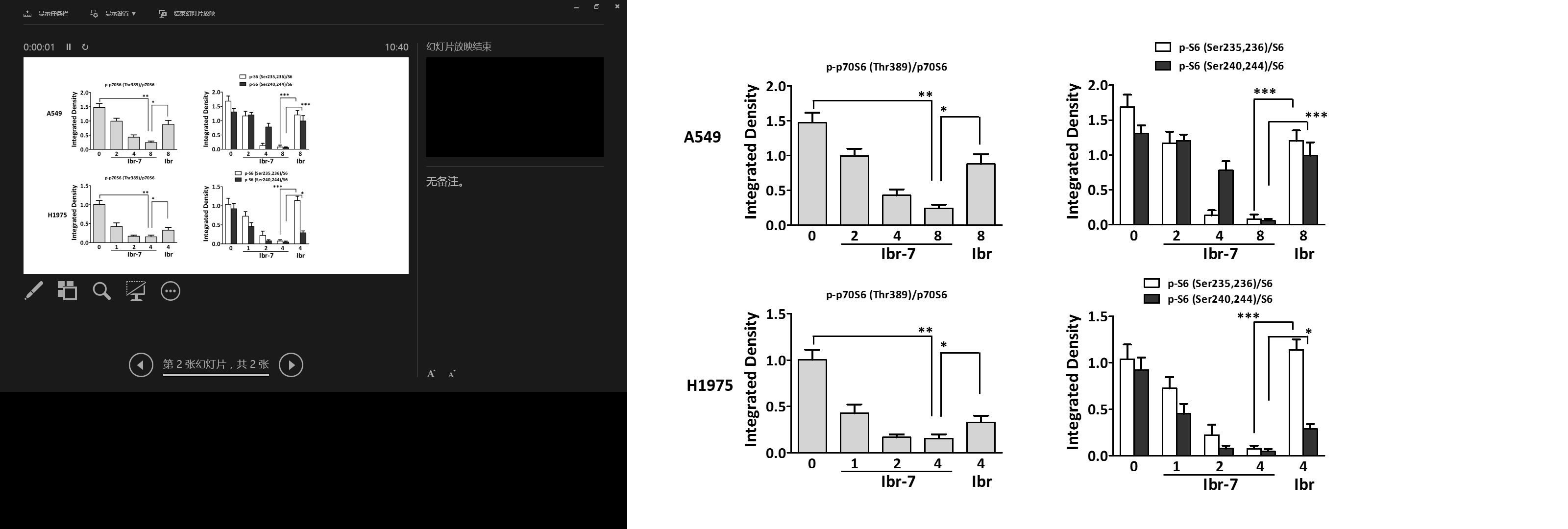


Figure S6. Active p-S6 overexpression slightly affect Ibr-7’s anti-proliferation effect. A549 cells were transfected with active p-S6 (235/236/240/244) (rpS6 regulates blood–testis barrier dynamics through Akt-mediated effects on MMP-9. Journal of Cell Science, 2014, 127, 4870-4882.) for 8 h and treated with Ibr-7 for another 8 h before western blotting assay. Cell viability was determined after cells were treated with Ibr-7 for 48 h. Three independent experiments were performed, and data were presented as mean ± SD. * p < 0.05


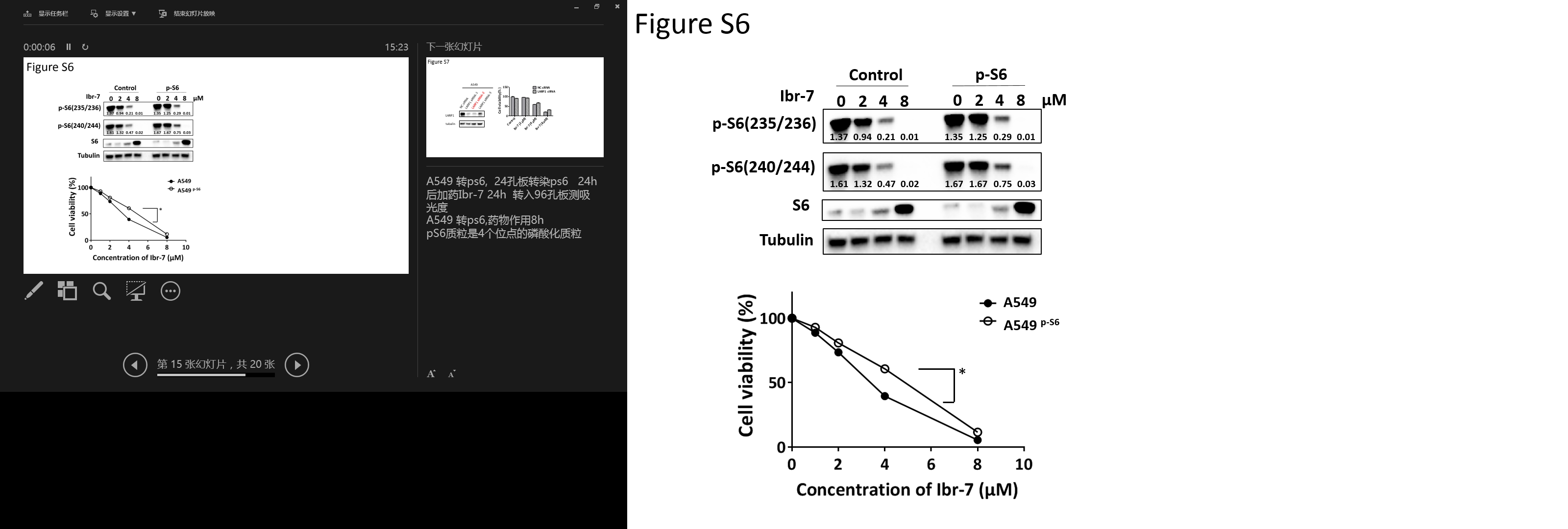


Figure S7. Knockdown of EGFR had negligible effects on Ibr-7’s anti-proliferation effect. **A,** EGFR in A549 cells were silenced by siRNA transfection. EGFR siRNA-3 was selected for CCK-8 assay and western blots. **B,** cells were treated with Ibr-7 for 48 h before CCK-8 assay. C, western blots were performed to analyze the protein expression after the eradication of EGFR under the exposure to either Ibr-7 or Ibr (Ibrutinib). Three independent experiments were performed, and data were presented as mean ± SD. n.s. = non-significant.


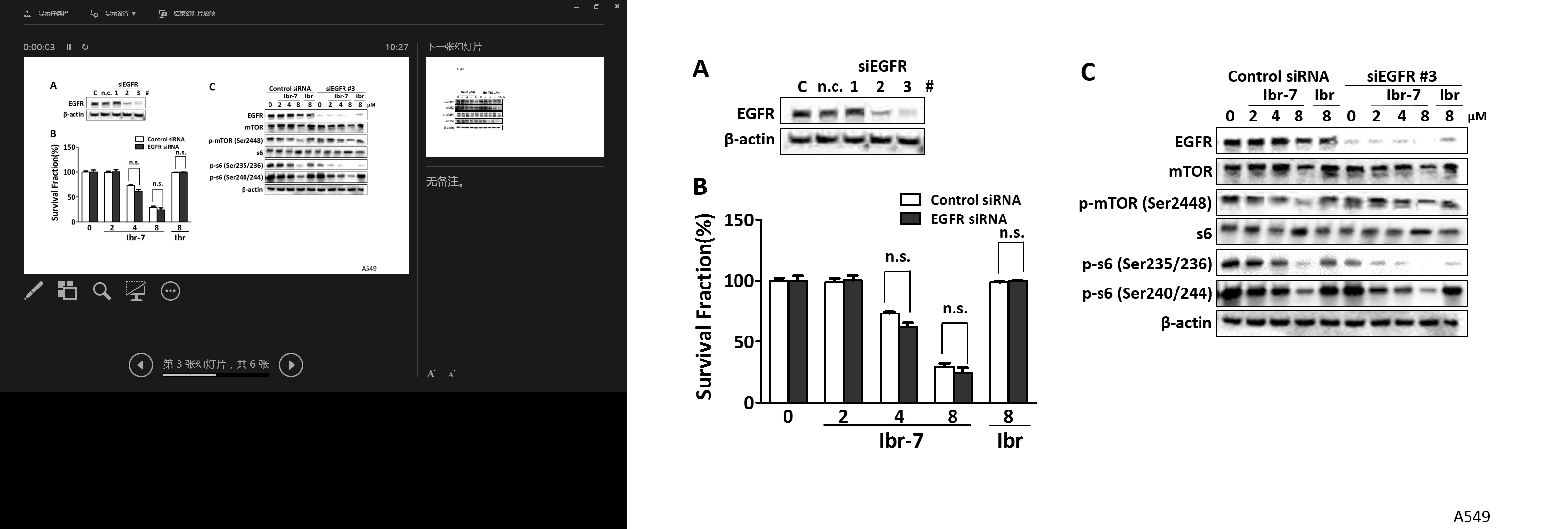


Figure S8. Knockdown of LARP1 did not undermine Ibr-7’s anti-proliferation effect. LARP1 in A549 cells were silenced by siRNA transfection. LARP1 siRNA-2 was selected for the viability assay. Cells were treated with Ibr-7 for 48 h before CCK-8 assay. Three independent experiments were performed, and data were presented as mean ± SD.


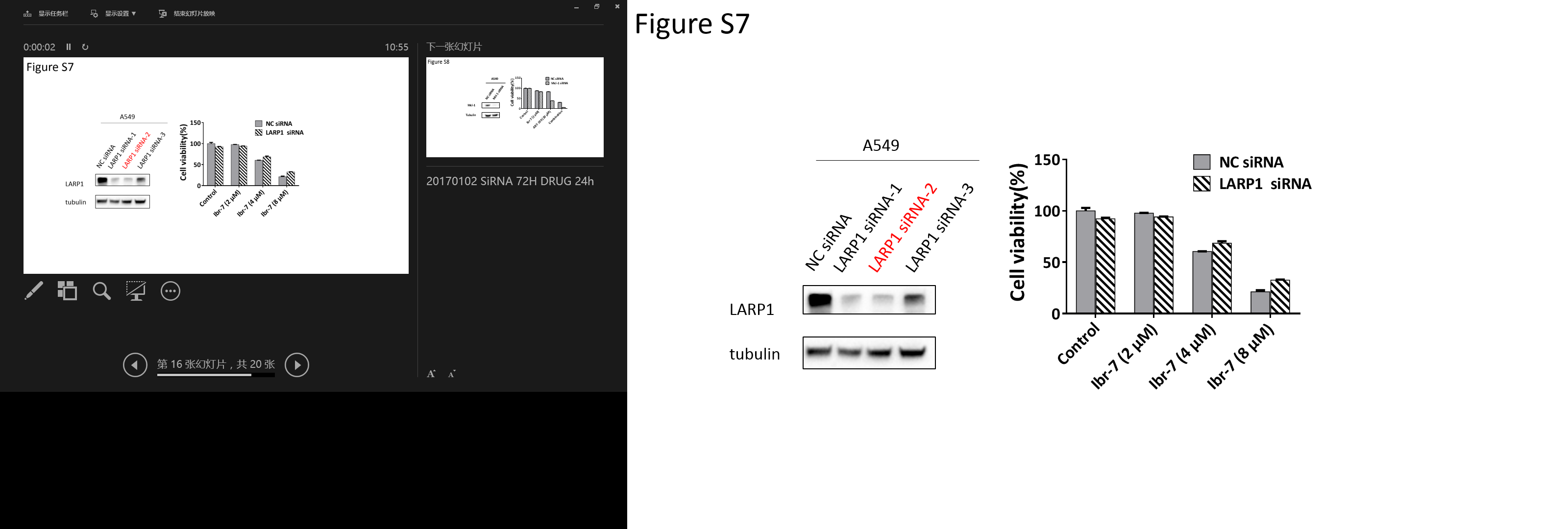


Figure S9. Mcl-1 played a key role in the antitumor effect of ABT-199 and combination treatment. Mcl-1 was silenced by siRNA in A549 cells. Cells were treated with Ibr-7, ABT-199 or the combination for 48 h before CCK-8 assay. Three independent experiments were performed, and data were presented as mean ± SD.


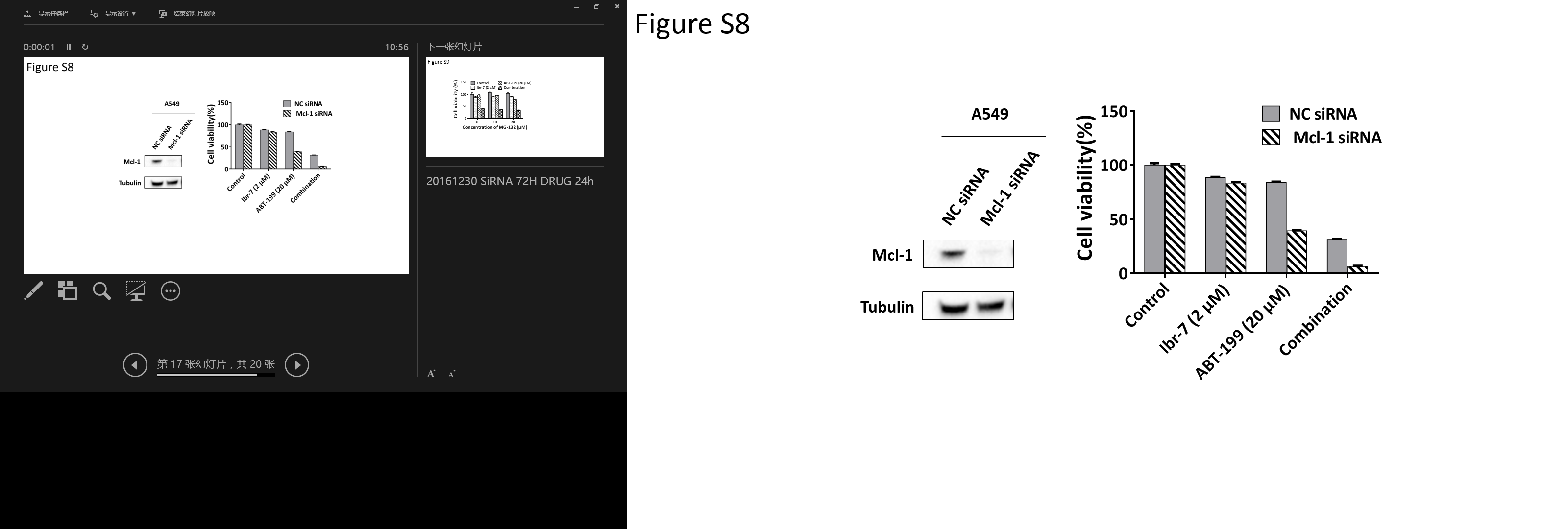


Figure S10. MG-132 showed no cytotoxicity in A549 cells. A549 cells were treated with Ibr-7, ABT-199 or the combination and co-cultured with MG-132 for 24 h before CCK-8 assay. Three independent experiments were performed, and data were presented as mean ± SD.


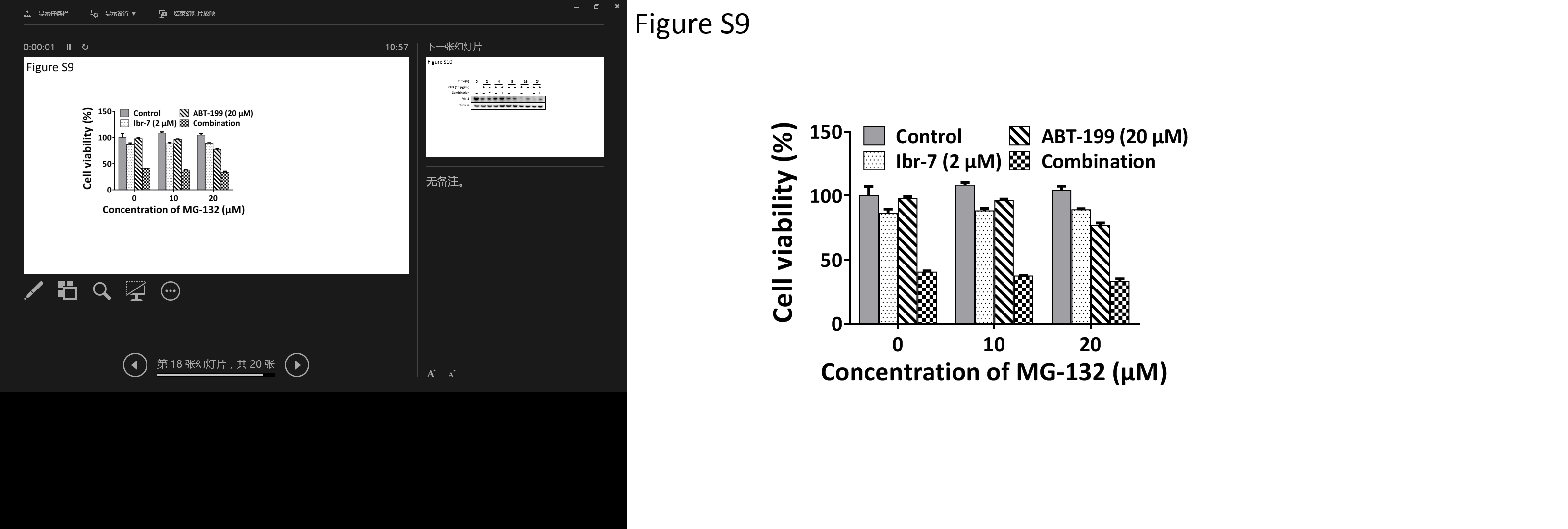


Figure S11. CHX did not expedited the degradation of Mcl-1. A549 cells were co-incubated with CHX and the combination treatment of ABT-199 (20 μM) and Ibr-7 (2 μM) for different times before western blotting assay.


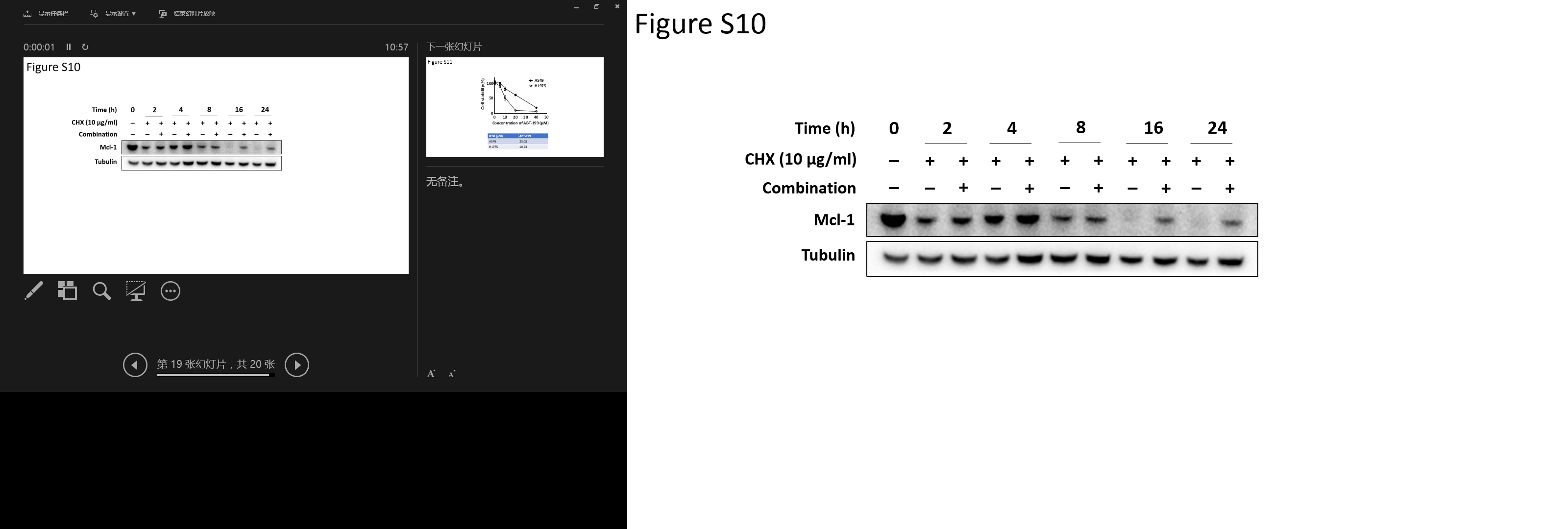


Figure S12. The cytotoxicity of ABT-199 on A549 and H1975 cells. Cell were treated with ABT-199 for 48 before CCK-8 assay. Three independent experiments were performed, and data were presented as mean ± SD.


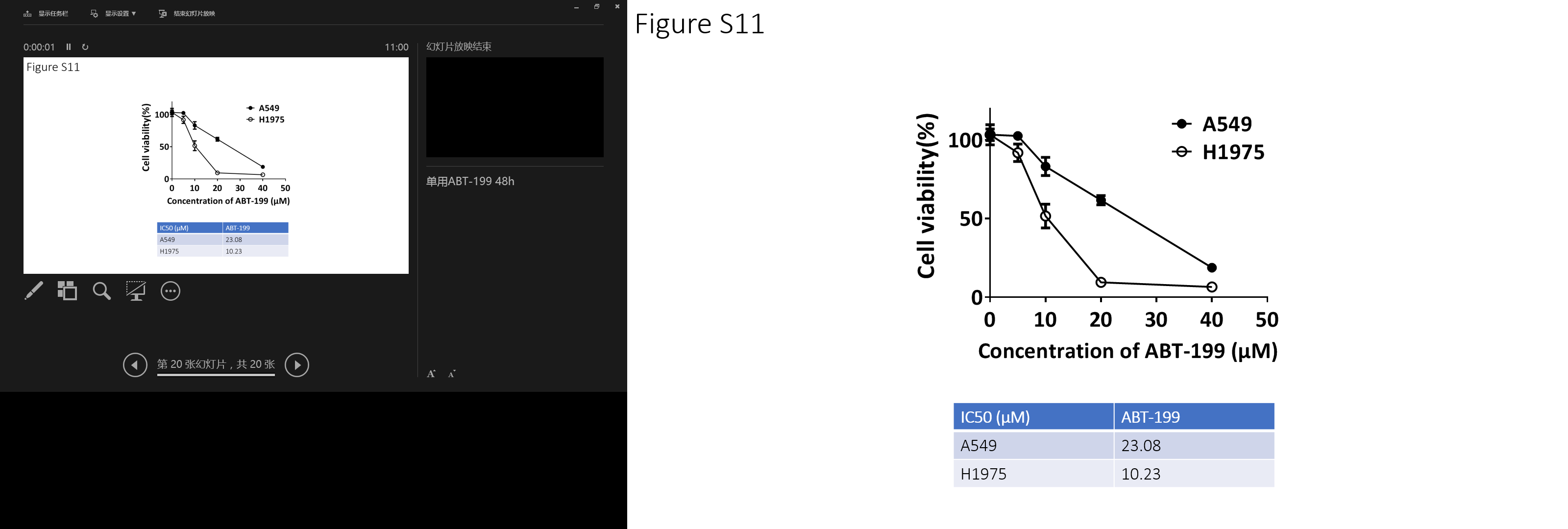

Supplement: Supplementary file 1 — Table S1. The IC50 values of ibrutinib and Ibr‐7 against various cancer cell lines. Table S2. Cell viability of 15 primary lung cancer cells after treatment with 4 μm of compounds for 24 h, and cultured for another 120 h before neutral red stain and fixation. Table S3. PK parameters of Ibr‐7 in SD rat (intragastric 30 mg·kg−1). Table S4. The inhibitory activity of ibrutinib and Ibr‐7 on five kinases. Fig. S1. DAPI stain of cell nucleus. Fig. S2. Bodyweights of xenograft nude mice after administration of Ibr (ibrutinib) or Ibr‐7, 60 mg·kg−1 twice a day. Fig. S3. Western blotting assay of EGFR and p‐EGFR. Fig. S4. Western blotting assay of p‐ErbB‐2, ErbB‐2, p‐ErbB‐4 and ErbB‐4. Fig. S5. Quantitative analysis of proteins. Fig. S6. Active p‐S6 overexpression slightly affects the anti‐proliferation effect of Ibr‐7. Fig. S7. Knockdown of EGFR had negligible effects on the anti‐proliferation effect of Ibr‐7. Fig. S8. Knockdown of LARP1 did not undermine the anti‐proliferation effect of Ibr‐7. Fig. S9. Mcl‐1 played a key role in the antitumor effect of ABT‐199 and combination treatment. Fig. S10. MG‐132 showed no cytotoxicity in A549 cells. Fig. S11. CHX did not expedite the degradation of Mcl‐1. Fig. S12. The cytotoxicity of ABT‐199 on A549 and H1975 cells. [file MOL2-13-946-s001.docx]
